# Modulation of *Drosophila* Retinal Epithelial Integrity by the Adhesion Proteins Capricious and Tartan

**DOI:** 10.1371/journal.pone.0001827

**Published:** 2008-03-19

**Authors:** Yanlan Mao, Martin Kerr, Matthew Freeman

**Affiliations:** MRC Laboratory of Molecular Biology, Cambridge, United Kingdom; Katholieke Universiteit Leuven, Belgium

## Abstract

**Background:**

The development of the *Drosophila* eye imaginal disc requires complex epithelial rearrangements. Cells of the morphogenetic furrow are apically constricted and this leads to a physical indentation in the epithelium. Posterior to the furrow, cells start to rearrange into distinct clusters and eventually form a precisely patterned array of ommatidia. These morphogenetic processes include regulated changes of adhesion between cells.

**Methodology/Principal Findings:**

Here, we show that two transmembrane adhesion proteins, Capricious and Tartan, have dynamic and complementary expression patterns in the eye imaginal disc. We also describe novel null mutations in *capricious* and double null mutations in *capricious* and *tartan*. We report that they have redundant functions in regulating the architecture of the morphogenetic furrow and ommatidial spacing.

**Conclusions/Significance:**

We conclude that Capricious and Tartan contribute to the adhesive properties of the cells in the morphogenetic furrow and that this regulated adhesion participates in the control of spacing ommatidial clusters.

## Introduction

The development of the *Drosophila* compound eye is a complex process involving the interplay of many signalling pathways (reviewed in [1]). The *Drosophila* eye is composed of a regular hexagonal lattice of about 800 individual facets known as ommatidia. Each ommatidium consists of a unit of eight photoreceptor neurons (R1–R8) and four cone cells, and is surrounded by pigment cells. The eye develops from a monolayer epithelium known as the eye-antennal imaginal disc. At the start of the third larval instar, the cells in the imaginal disc start to differentiate. This differentiation starts at the posterior of the disc and sweeps anteriorly, preceded by a physical indentation known as the morphogenetic furrow (MF). Developing rows of ommatidia are left in its wake, and this progressive development implies that there is a gradient of developmental stages in a single disc, with the most mature being at the posterior [2].

Most of the cells in the eye disc have a columnar epithelial morphology, but in the morphogenetic furrow they become apically constricted, reviewed in [3]. As a result of this constriction, these cells change from being columnar to bottle-shaped and the consequent change in epithelial packing produces the indentation of the furrow itself [4]. Immediately after the passage of the furrow, and therefore posterior to it, cells begin to rearrange, developing from random packing into first lines of cells, then arcs, and finally morphologically distinct clusters within the epithelium. This process depends on myosin II contractility [4] but presumably also requires precise changes in the adhesive properties of cells as the clusters separate from their neighbours. In fact, adhesive changes can be directly observed–the clusters show increased levels of apical Armadillo/β-catenin, a key component of the adherens junctions, a phenomenon dependent on Atonal and the epidermal growth factor receptor (EGFR) pathway [5]. Beyond this increase in adherens junctions, little is known about the adhesion processes that participate in the clustering process.

Capricious (Caps) and Tartan (Trn) are highly similar transmembrane proteins with multiple extracellular leucine rich repeats (LRRs) and shorter intracellular domains [6], [7]. They share 67% protein sequence identity in their extracellular domains, which consist of 14 LRR repeats, but only 15% overall identity in their intracellular domains, including a conserved motif of 31 amino acids adjacent to the membrane. Since they lie within 115 kb of each other in the genome, it is likely that they represent a relatively recent gene duplication event. Although their exact molecular function is not well characterised, they can act as homotypic adhesion proteins in cell culture [8] and at least in some contexts their intracellular domains are dispensable [9], [10], supporting the idea that their primary roles are in cell adhesion. Consistent with this, their functions have mostly been associated with their adhesion properties. Caps is required for targeting a subset of embryonic motor neurons to their specific muscles during embryonic development [7], [10] and in targeting R8 photoreceptor axons to the appropriate layers of the optic lobe [8]. Caps and Trn have also been implicated in the formation of affinity boundaries between dorsal and ventral compartments in the developing wing imaginal disc [9], [11], [12], [13]. Very recently they have been shown to have overlapping functions in adhesion of cells in the developing leg imaginal disc [14].

As described above, the developing eye imaginal disc undergoes morphological plasticity as it differentiates, and this involves precisely ordered remodelling of epithelial cell contacts [4]. Here we describe the specific and complementary expression patterns of Caps and Trn in the imaginal eye disc and their redundant roles in regulating aspects of epithelial organisation in the morphogenetic furrow and the spacing of developing ommatidia.

## Results

### Dynamic and complementary expression pattern of *capricious* and *tartan* in the eye

**Figure 1:**
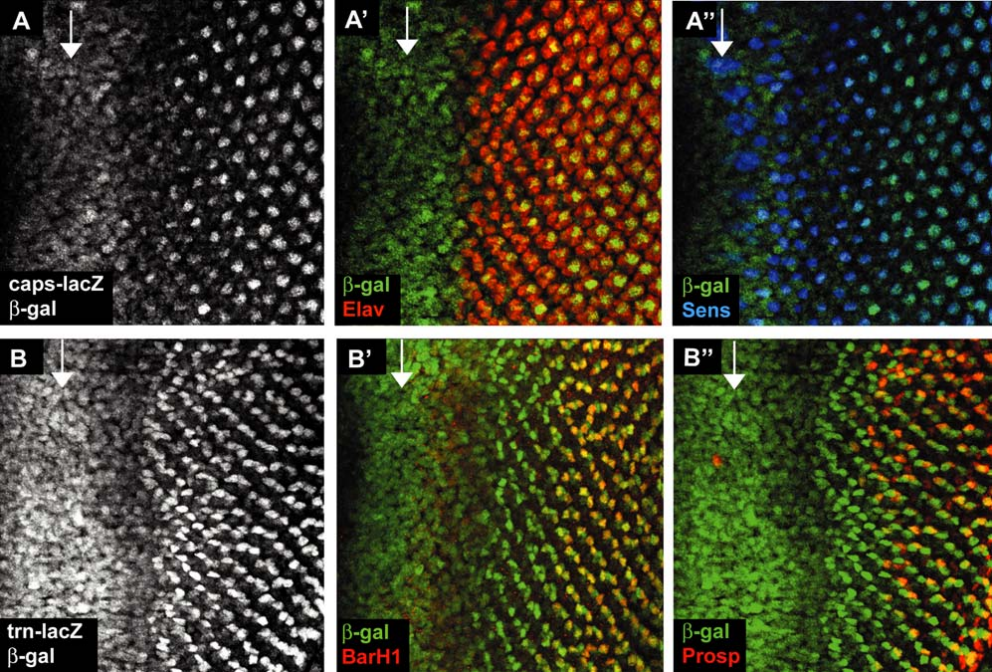
* caps* and *trn* expression in the eye. The arrow in each panel marks the morphogenetic furrow (MF) and anterior is to the left in all images, unless otherwise stated. (A) *caps-lacZ* expression in 3^rd^ instar eye disc. Staining with anti-β−gal revealed *caps-lacZ* expression in the furrow and in subsets of cells after the furrow. (A'–A”) Co-staining with anti-Elav (a photoreceptor marker) and anti-Senseless (R8 specific marker) identified the cells eventually expressing high levels of *caps-lacZ* as photoreceptor R8. (B) *trn-lacZ* expression in 3^rd^ instar eye disc. Staining with anti-β−gal revealed *trn-lacZ* expression in the furrow and in a different subset of cells from *caps-lacZ* after the furrow. (B'–B”) Co-staining with anti-BarH1 (R1 and R6 specific marker) and anti-Prospero (R7 and cone cell marker) identified R1, 6 and 7 as the photoreceptors expressing high levels of *trn-lacZ*.

We initially identified an allele of *caps* in a screen for modifiers of EGF receptor signalling in the eye. This interaction proved inconsistent and was not supported by other alleles of *caps*, so we have not pursued this further. We noticed, however, that *caps* and *trn* have developmentally regulated expression patterns in the eye. In 3^rd^ instar eye imaginal discs, *caps-lacZ* is expressed in all cells in the morphogenetic furrow (arrow Fig. 1A) and at a lower level in cells just posterior to the furrow before becoming restricted to single photoreceptor cells (Fig. 1A'). By simultaneous staining with the R8 photoreceptor marker Senseless [15], we showed that the single cells eventually expressing *caps-lacZ* are the R8 cells, the founders of ommatidial development (Fig. 1A”). This result is consistent with the expression pattern reported by Shinza-Kameda et al. (2006), although they limited their description to the later stages when *caps* is restricted to R8. *trn* is a close sequence relative of *caps* and in the wing imaginal disc they are believed to act in partnership as adhesion proteins that regulate cell affinity at compartment borders [11]. We therefore examined the expression pattern in the eye of *trn-lacZ.* Interestingly, *trn-lacZ* is also expressed dynamically, initially in all cells in the furrow, then at a lower level in cells just posterior to the furrow, before becoming restricted to a non-overlapping subset of photoreceptor precursors from *caps* (Fig. 1B). *trn-lacZ* colocalised with R1 and R6 markers anti-BarH1 [16] and R7 marker anti-Prospero [17] thereby identifying the cells as R1, 6 and 7 (Fig. 1B' and 1B”). In summary, both *caps* and *trn* are widely expressed in the morphogenetic furrow, and each then becomes restricted to non-overlapping subsets of photoreceptors. These complementary expression patterns in the eye suggested that the Caps and Trn proteins might have a previously unrecognised function in eye development.

### Localisation of Capricious and Tartan proteins in the developing eye

**Figure 2:**
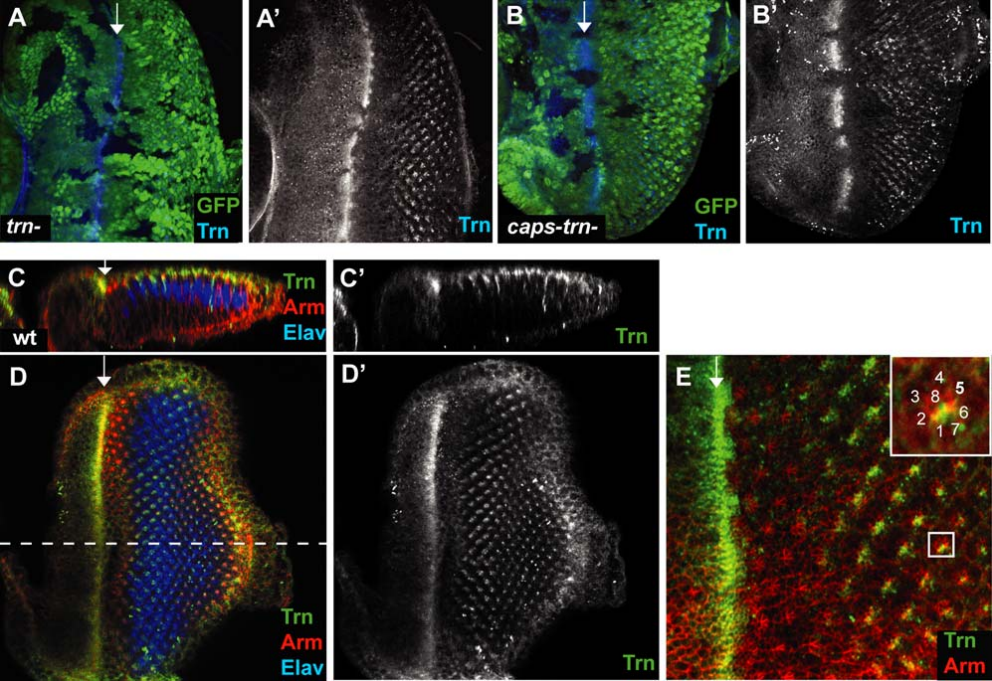
Localisation of Tartan protein in the eye. (A–A') The Trn antibody recognised endogenous levels of Trn in the 3^rd^ instar eye disc. The signal is absent in clones of *trn^28.4^* null cells (marked by loss of GFP, green), confirming antibody specificity. (B–B') The Trn antibody signal was also lost in *caps^Del1^trn^28.4^* double null clones, marked by loss of GFP (green). (C–C') Z-section along the A-P axis of a wild type disc stained with anti-Trn. Trn is expressed only in the anterior half of the furrow and mostly in the apical surface of photoreceptors, as well as in some basolateral membranes. (D–D') Apical planar views of the corresponding discs in (C–C'). Trn is expressed in the furrow and in subset of photoreceptors after the furrow. The dashed line indicates the position of the sagittal section in C and C'. (E) Enlarged view of the disc in D. The inset shows an enlarged view of the marked ommatidium with the positions of each photoreceptor labelled. Trn expression is located in the expected positions of R1, 6, 7.

We raised specific antibodies against Caps and Trn to examine their expression pattern in more detail. Unfortunately the Caps antiserum did not reliably detect the endogenous level of Caps protein in eye discs. In contrast, the Trn antibody successfully recognised Trn protein in wild-type discs. Its specificity was confirmed by the loss of signal in clones of cells mutant for Trn (Fig. 2A and 2B) but not in clones of cells mutant for Caps (data not shown). The antibody staining pattern confirmed the *caps-lacZ* expression pattern: Trn is expressed broadly in the morphogenetic furrow and in subsets of ommatidial cells after the furrow (Fig. 2D). As photoreceptor specific markers are almost all nuclear, and Trn is membrane localised, overlapping staining patterns cannot readily be used to confirm the identity of the specific ommatidial cells stained posterior to the furrow. However, the expression pattern is fully consistent with that of the *trn-lacZ* line, that is, the staining is localised in the expected location of R1, 6 and 7 but not of R8, 2, 5, 3, 4 (Fig. 2E, see inset). Z-sections along the anterior-posterior axis of the disc revealed that it is expressed mostly in the apical membrane of photoreceptor cells but is also visible in some basolateral membranes. Interestingly, Tartan is only expressed in the anterior half of the furrow (Fig. 2C).

### Caps and Trn single mutants have no affect on eye development

**Figure 3:**
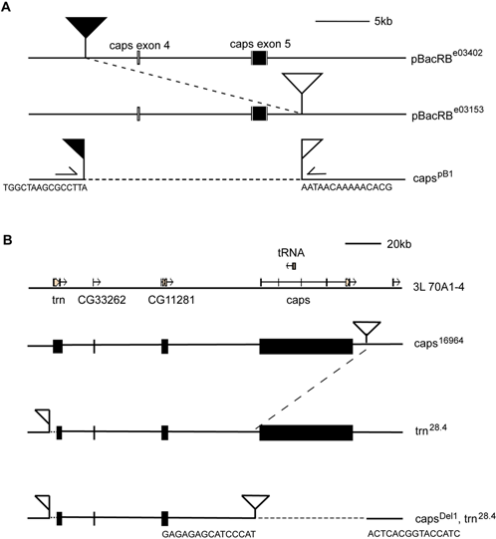
Generation of *caps* null and *caps trn* double null mutants. (A) Generating *caps^pB1^* null. Two *piggyBac* element insertion lines were used for FLP-FRT based recombination to delete the entire coding sequence of *caps*, which is contained in exon 5. *pBacRB^e03402^* is inserted upstream of exon 4 and *pBacRB^e03153^* is inserted downstream of exon 5. Upon heatshock, recombination occurs between these two *piggyBac* elements, deleting the intervening region, regenerating a complete *piggyBac* element from half of each of the original *piggyBacs*. The deletion sites were confirmed by using genomic primers (marked) outside the *piggyBac* elements to amplify across the newly formed element (about 6 kb). Sequencing outward from either end of the PCR fragment identified the precise deletion sites. Flanking sequence of the new deletion is shown. (B) Generating *caps^Del1^trn^28.4^* double null. *caps^16964^* has a GS element inserted downstream of the *caps* gene. *trn^28.4^* is a null allele of tartan generated by P-element excision. *caps^16964^* was used to induce male recombination with the *trn^28.4^* chromosome. This allowed the simultaneous deletion of the *caps* gene and recombination onto the existing *trn^28.4^* null chromosome. The GS element remains intact after the recombination allowing its precise new position, and any deletions, to be verified by inverse PCR and sequencing. The entire *caps* gene was deleted, but no other gene (apart from one tRNA gene) was affected. Flanking sequence of the new deletion is shown.

**Figure 4:**
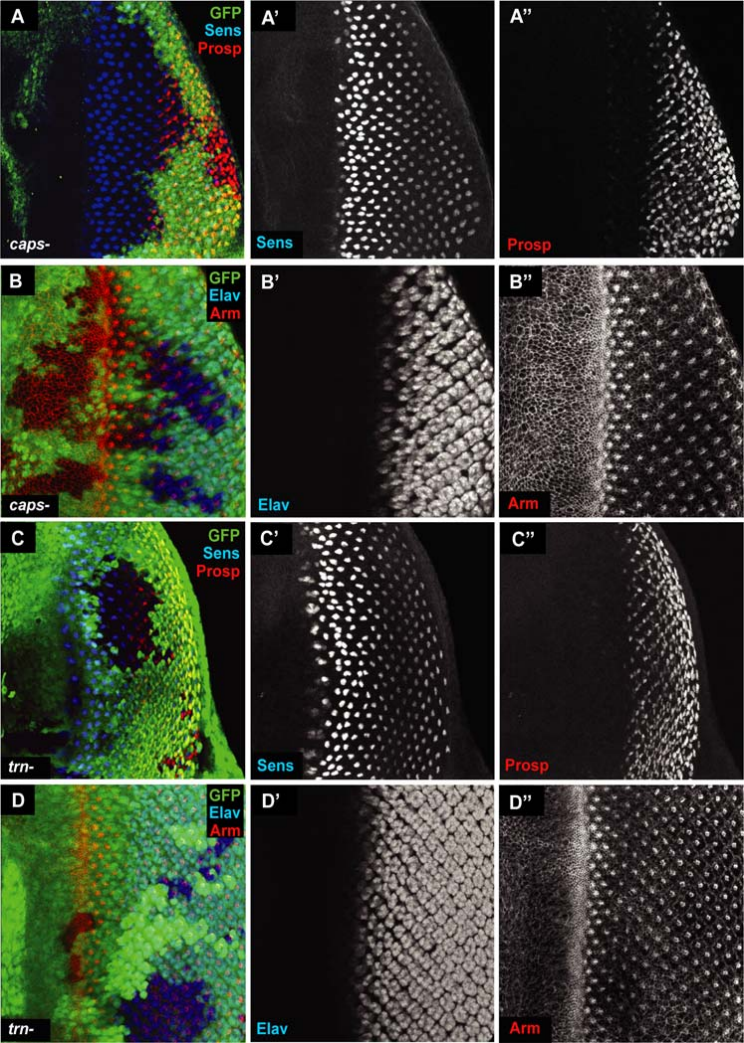
* caps* and *trn* single null clones. (A–B) *caps^pB1^* clones in the 3^rd^ instar eye disc. Mutant tissue is marked by lack of GFP (green). Anti-Senseless (Sens), is used to identify R8 cells (A') and anti-Prospero (Prosp) is used to identify R7 and cone cells (A”). Anti-Elav marks all photoreceptor cells (B') and anti-Armadillo (Arm) marks the adherens junctions of cells, thereby outlining all cells (B”). No defects could be detected in *caps^pB1^* mutant tissue. (C–D) *trn^28.4^* clones in the 3^rd^ instar eye disc. Mutant tissue is marked by lack of GFP (green). As with *caps^pB1^* clones, no defects were observed.

The expression of Caps and Trn suggested that they might participate in retinal development. When this work was initiated, there were no *caps* null mutations reported: the strongest hypomorph, *caps^65.2^*, still retained 10–20% normal expression level [7], [8]. We therefore made a null mutant of *caps* by targeted recombination induced deletion between two *piggyBac* elements (see Materials and Methods). This allele, *caps^pB1^,* was designed to delete exons 4 and 5 of the *caps* gene (Fig. 3A); exon 5 contains the whole coding sequence. The mutation was confirmed by PCR and sequencing (sequences flanking deletion site are shown in Fig. 3A). Previously described *caps* mutations are embryonic lethal [7] and, as expected, *caps^pB1^* was also lethal. Mitotic recombination was therefore used to generate loss-of-function clones in the developing eye. These were induced in *Minute* and non-*Minute* backgrounds [18], [19], allowing the production of a full range of clone sizes. Development within these clones (marked by lack of GFP, green) appeared normal, and a variety of antibodies against cell-type specific markers, including Senseless (R8-specific), Prospero (R7) and Elav (all photoreceptors) [20], were expressed in indistinguishable patterns from wild-type discs and from adjacent wild-type tissue (Figure 4A'–B'). In particular, R8 cells, where Caps is expressed (Fig. 4A'), appear normal, and are correctly spaced in null mutants. Since Caps is an adhesion protein and is expressed strongly in the furrow, prior to axonal outgrowth, we focused carefully on the morphology of cells in the furrow and the spacing of ommatidial clusters immediately posterior; these were also unperturbed in *caps^pB1^* clones (Fig. 4B”).

We also made clones of a null allele of *trn*, *trn^28.4^*. Mutant clones (marked by lack of GFP, green) also had no defects (Figs. 4C and D). Again, photoreceptor markers Elav, Senseless and Prospero appeared wild type, and no defects in the furrow or in ommatidial spacing could be detected.

### *caps* and *trn* double mutant affects apical constriction of cells in the furrow

**Figure 5:**
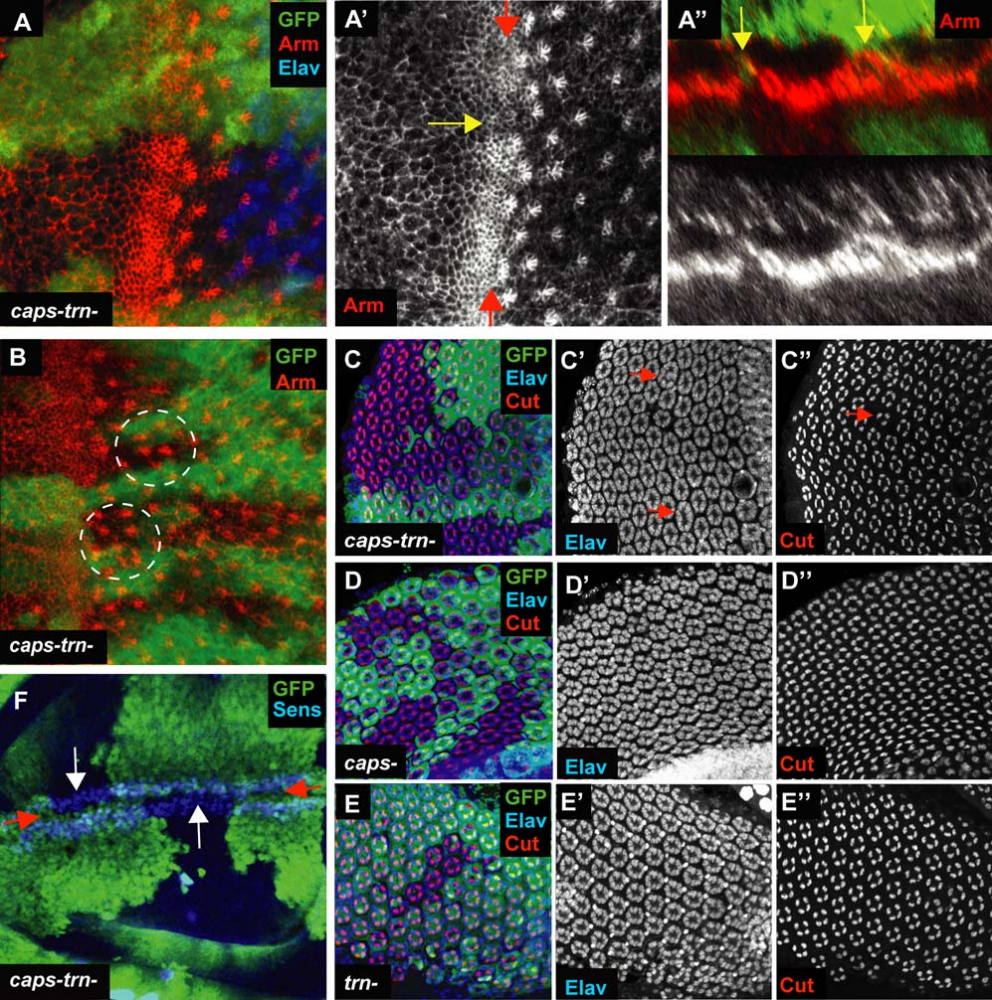
* caps trn* double null clones. (A) *caps^Del1^ trn^28.4^* double null clones in the 3^rd^ instar eye disc. Mutant tissue is marked by lack of GFP (green). Anti-Elav marks all photoreceptors. Anti-Armadillo (Arm) marks the apical surface of cells and is accumulated at a high level in the apically constricted cells of the furrow. At the border between wild type and *caps^Del1^ trn^28.4^* tissue, there is a reduction in Arm accumulation in the cells of the furrow and the apical surface of cells is expanded (yellow arrow A'). (A”) A Z-section along the furrow (between the two red arrows in A'). At the clone boundaries, cells are taller in the apical-basal direction, producing ‘bumps’ in the furrow (two yellow arrows). (B) A *caps^Del1^trn^28.4^* clone (marked by lack of GFP, green) where ommatidia near the clone boundaries are mis-positioned (circled in white). (C) *caps^Del1^trn^28.4^* clones in pupal retinae (marked by lack of GFP, green). Elav is used to mark the photoreceptors and Cut is used to mark cone cells (four per ommatidium) [27]. At mutant-wild type borders neighbouring ommatidia sometimes fuse with each other (red arrows in C'). Correct cone cell numbers are also sometimes disrupted (red arrow in C”). (D) *caps^pB1^* clones in pupal retinae (marked by lack of GFP, green). No defects in mutant tissue or clone boundaries can be seen. (E) *trn^28.4^* clones in pupal retinae (marked by lack of GFP, green). Again, the retinae are phenotypically wild type. (F) *caps^Del1^trn^28.4^* clones in the wing (marked by lack of GFP, green) do not visibly affect the DV boundary (between the two red arrows), as marked by anti-Senseless (Sens). Clones do not cross the DV boundary (white arrows).

Since *caps* and *trn* single mutants did not affect eye development, and since Caps and Trn are highly related proteins, we wondered whether they might act redundantly in eye development. To address this, we generated a *caps trn* double null mutation, *caps^Del1^ trn^28.4^*, by using P-element induced male recombination [21] to simultaneously delete *caps* and recombine the new mutation onto the existing *trn^28.4^* null allele (see Figure 3B and Materials and Methods for details). This double *caps^Del1^ trn^28.4^* null retains the intervening genes *CG33262* and *CG11281*, so represents a ‘clean’ removal of the two related proteins. Mitotic clones of the *caps^Del1^ trn^28.4^* mutation (marked by lack of GFP, green) showed subtle but consistent defects. Within the morphogenetic furrow, the mutant cells showed normal levels of apical constriction, and accumulated high levels of Armadillo/β-catenin indistinguishably from the wild type. However, at the clone border between mutant and wild-type cells, there was a consistent reduction in the apical constriction of cells and their Armadillo accumulation in adherens junctions (Fig. 5A' yellow arrow). This phenotype is fully penetrant but appears more pronounced when the clone boundary is perpendicular to the morphogenetic furrow.

The apical constriction of morphogenetic furrow cells generates the indentation of the furrow itself [4]. Sagittal sections in the Z-axis of *caps^Del1^ trn^28.4^* clones along the furrow (between the two red arrows in Fig. 5A'), showed that the cells at the clone boundary with the enlarged apical profiles were also taller than their neighbours, that is, their apical surfaces were elevated, thereby disrupting the furrow itself (arrows, Fig. 5A”).

These related phenotypes of relaxation of apical constriction and increase in apical-basal height of the cells were a non-autonomous effect: they were observed in both mutant and wild-type cells at the clone boundary. The range of the phenotype was only 2–3 rows of cells beyond the clone border, and in some cases this non-autonomy was predominantly in the wild-type, and sometimes predominantly in the mutant territory. Given the subtle nature of the effects, we re-examined the borders of clones of single mutants for *caps* or *trn* but confirmed that they were never visibly affected.

### Caps and Trn double mutant perturbs ommatidial spacing

A second phenotype associated with the *caps^Del1^ trn^28.4^* double null clones (marked by lack of GFP, green) was a perturbation in ommatidial spacing in third instar eye discs (Fig. 5B, circled). Again, this was only apparent at the boundaries between mutant and wild-type cells. Ommatidia close to these boundaries were often clearly displaced from their normal positions but there was no obvious change to their individual morphology, nor was the total number of ommatidia obviously affected. 25 individual eye discs containing clones were analysed, and the number of ommatidia adjacent to clonal boundaries was counted, along with the number of these ommatidia that were displaced from their normal position. In total, of 846 ommatidia at boundaries, 187 (i.e. 22%) were displaced. This phenotype is also non-autonomous, with both mutant and wild type ommatidia showing mis-positioning. These spacing defects remain later, at pupal stages of eye development, and they are made more apparent by the fusion of neighbouring ommatidia that are abnormally close to each other (Fig. 5C). These fusions are observed in about 5% of ommatidia adjacent to clone boundaries. We also observed very occasional defects in the normal number of cone cells (e.g. arrow in Fig. 5C”). As with the third instar eye disc, the pupal phenotypes were not observed in clones mutant for *caps* or *trn* alone (Figs. 5D and 5E).

Since we observed defects only at the boundaries between *caps^Del1^ trn^28.4^* mutant and wild type tissue, we wondered whether the sudden step-like changes in Caps and Trn levels were more important than the overall levels of these adhesion proteins. We therefore made clones over-expressing Caps and Trn but they did not show any visible furrow or ommatidial spacing defects at the clone boundaries (data not shown). This implies that the boundary effects seen in clones of *caps^Del1^ trn^28.4^* cells are caused by the juxtaposition of cells expressing Caps and Trn with cells not expressing them.

In summary, we conclude that there is a redundant function for Caps and Trn in controlling aspects of cell morphology and ommatidial spacing in *Drosophila* retinal development.

### *caps* and *trn* double mutant in the wing

One of the main tissues in which Caps and Trn have been studied is the developing wing imaginal disc. Despite evidence that *caps* and *trn* have an important function in maintaining compartment borders in the wing, previously studied mutants in these genes have not affected the dorsal-ventral boundary [11]. We therefore took advantage of having made a previously unavailable double null mutation to look at DV border formation in the wing. Clones of *caps^Del1^trn^28.4^* double null (marked by lack of GFP, green) did not perturb or cross the boundary, as marked by staining with an antibody against Senseless (Fig. 5F, white arrows). This is consistent with earlier data, where clones of cells simultaneously null for *trn* and hypomorphic for *caps* did not cause defects at the DV boundary [11]. The fact that complete loss of both proteins does not affect DV boundary formation or maintenance suggests that Caps and Tartan are not essential for compartmentalising cells in this part of the wing.

## Discussion

We have shown that the related adhesion proteins Capricious and Tartan have redundant functions in the remodelling of epithelial cell contacts that occur during the early stages of *Drosophila* eye development. Each is expressed in a two phase pattern, first broadly in the furrow and then later in non-overlapping subsets of photoreceptors: Caps in R8 and Trn in R1, 6 and 7. We have made a null mutation of *caps* and also a double null, in which both *caps* and *trn* are absent. Analysis of these mutations shows that while removal of either gene alone has no phenotype, the loss of both leads to subtle but reproducible defects in retinal development. The earliest phenotype is a reduction of apical constriction and accumulation of Armadillo in the morphogenetic furrow. Slightly later, we see displacement of ommatidia from their normal very precise array. Finally, this displacement leads to occasional fusion of neighbouring ommatidia and other minor defects in the pupal retina. Intriguingly, all the defects we observe are limited to clone boundaries; cells fully within the mutant clones appear normal.

The *caps^Del1^trn^28.4^* phenotypes in the eye are relatively minor. They are nevertheless reproducible and quite penetrant. Essentially all clones that cross the furrow perpendicularly show a reduction in apical constriction and Armadillo staining at their clone boundaries, and 22% of ommatidia that lie at the clone boundary are detectably misplaced; ommatidial fusion defects in the pupal retina are rarer, at about 5%. We propose that these phenotypes are all a consequence of the initial furrow defects, and that these are caused by loss of the furrow expression of Caps and Trn. This implies that the later, photoreceptor-specific expression of Caps and Trn does not participate in the phenotypes reported here. This proposal is based on the following logic. First, the redundant function of the two proteins is difficult to reconcile with non-overlapping expression: if they are in different cells, how can they replace each other's function? Although it would be possible to imagine a scenario where this could occur, a more parsimonious explanation is that the redundant phenotype depends on their function where they are co-expressed, in the furrow. Second, the expression of Caps in R8 is already known to have a quite separate function, in the targeting of the R8 axon growth cones to the appropriate layer of the optic lobe [8]. The R8 cell bodies are in the retina, which is why we see *caps-lacZ* expression there, but the protein must be transported to the axon terminals. Our discovery of an equally specific but non-overlapping expression of Trn, suggests that it too might have an analogous function in axon targeting, although this prediction has not been tested.

The idea that the later, photoreceptor specific expression of Caps and Trn is responsible for axonal guidance defects, but not retinal patterning, appears inconsistent with the protein expression of Trn that we see at the apical surface of the photoreceptors, i.e. in the retina, distant from the axon terminals. Unfortunately, we could not detect the wild-type protein expression of Caps, which we know to be involved in axonal guidance, so it is possible that Caps protein is localised very differently from Trn–only in the axons. Although we must await a better anti-Caps antibody to resolve this fully, on balance we suspect that the apical expression of Trn, and possibly Caps, either reflects a function distinct from the retinal defects we report here and also from axonal guidance; or that it is a non-functional consequence of the intracellular trafficking pathways that transport the functional pool to the axon terminal.

The third reason for suggesting that the functions we have uncovered are dependent on Caps and Trn in the furrow, and that the later defects in spacing are secondary consequences of a primary furrow defect is that this is consistent with the furrow acting to organise epithelial packing. Detailed inspection of cells in the furrow and immediately after they emerge from it, shows profound rearrangement that starts with straight lines of cells, evolving into arcs and finally into morphologically distinct clusters [4]. Adhesion defects in the furrow may disrupt this process such that ommatidial clusters and their spacing become less ordered. We do not understand why these phenotypes only manifest at clone boundaries, but we presume it is a consequence of a discontinuity in adhesive properties. Similarly, the short range non-autonomy of the phenotype is probably due to local cell packing problems caused by adhesion anomalies at the boundaries of wild-type and mutant tissue. Another possible explanation for the non-autonomous effects is that changes in cell shape and epithelial morphology in the furrow could affect the range or efficiency of intercellular signalling molecules, thereby affecting normal retinal development. Little is known about how epithelial characteristics can modulate secreted signals and this will be a fruitful area for future study.

A very recent paper by Sakurai et al. [14] has analysed the functions of Caps and Trn in the developing leg disc. They also show a completely redundant function caused by rather subtle adhesion defects. Leg disc development is, however, very different from eye development and the developmental consequences are therefore distinct. In the leg, the sharpening of a progressive border that develops between tarsus 5 and the pretarsus segment was compromised in double mutants. By analysing cell movement within the developing leg disc, Sakurai et al. proposed that Caps and Trn expression allows cell mobility within the epithelium: their downregulation coincides with reduced mobility, while their overexpression leads to cell invasion into inappropriate territories. In the eye, there is no evidence for significant mixing of cells within the epithelium and, as described above, our model suggests a different use of a rather similar function for these adhesion proteins. In both cases, however, Caps and Trn appear to regulate the ability of cells within an epithelium to reorganise with respect to their neighbours.

In summary, we interpret our results to imply that Caps and Trn expressed in the morphogenetic furrow participate in modulating the adhesivity of epithelial cells. At this stage in development, they are beginning to undergo complex and coordinated rearrangements, with concomitant adhesion changes with their neighbours. Even quite minor disruption of this process leads to alterations in epithelial packing that can have consequent effects on the spacing of ommatidia. The relatively minor retinal phenotype of loss of Caps and Trn implies that other adhesion proteins contribute to the overall regulation of this process. For example, *Drosophila* E-cadherin, an essential component of adherens junctions, is necessary for epithelial maintenance [22], and mutant (hypomorph) clones fail to form adherens junctions and lose their epithelial integrity completely. We suspect that complex regulation of adhesion may require the action of several adhesion systems. Our data also leads to the tentative suggestion that Trn may, like Caps, have a later function in photoreceptor neuron development, for example in axon targeting. Finally, and on a separate tack, our construction of a double null mutation for *caps* and *trn* allows us to show unambiguously that neither are essential for the normal formation of the dorsal-ventral boundary of the wing imaginal disc, a process that overexpressed Caps and Trn can disrupt [11].

## Materials and Methods

### *Drosophila* strains

*caps-lacZ* (*caps^02937^*) [7] and *trn-lacZ (trn^SO64117^)* [23] were obtained from Bloomington *Drosophila* Stock Centre.

*trn^28.4^*, a null allele generated by P element excision [6] was obtained from Allen Laughon. *hsflp; trn^28.4^FRT2A* [14] was obtained from Shigeo Hayashi.

*pBacRB^e03402^* and *pBacRB^e03153^* were obtained from Harvard Exelixis Stock Centre, and used for making the null *caps^pB1^* allele.

*caps^16964^* was obtained from the *Drosophila* Gene Search Project, Tokyo Metropolitan University.

### Mutagenesis/Mutant production

#### caps^pB1^

This new null allele of *caps* was made by exploiting the FLP-FRT based deletion strategy established by Exelixis [24], [25]. *piggyBac* elements *pBacRB^e03402^* and *pBacRB^e03153^* were used to delete exon 4 and the entire *caps* coding sequence in exon 5 (Fig. 3A). Upon heatshock, recombination occurs between these two *piggyBac* elements, deleting the region in between and replacing it with a reformed *piggyBac* element. The precise deletion sites were confirmed molecularly by using genomic primers (marked on Fig. 3A) outside the original *piggyBac* elements to PCR across the newly formed *piggyBac* element to obtain a diagnostic 6kb fragment (which would be about 20 kb in wild type). Sequencing the ends of the 6kb fragment identified the exact junction between genomic DNA and *piggyBac* DNA, and confirmed the exact deletion sites (genomic sequences shown in Fig. 3A). During the preparation of this manuscript, Sakurai et al. reported the isolation of an EMS induced null allele of *caps* [14].

#### caps^Del1^trn^28.4^

This new *caps trn* double null allele was made by P element-induced male recombination between the *trn^28.4^* null chromosome and a P element (*caps^16964^*) inserted 3′ of *caps* gene (Fig. 3B). The P-element induced recombination resulted in the deletion of the entire *caps* gene without disrupting any other gene apart from one tRNA gene that was also deleted. Double mutants were confirmed by non-complementation with *caps* and *trn* alleles and the exact deletion sites induced by the P-element were checked by inverse PCR and sequencing. The flanking genomic sequences are shown in Fig. 3B.

### Antibody production

Rabbit and guinea pig antibodies were raised against the intracellular domains of Caps and Trn, respectively. Caps antibody did not give a specific signal. Final bleed Trn antiserum was used at 1∶100 dilution to give a strong specific signal.

### Mosaic analysis

Mitotic clones in the eye and wing discs were induced by the FLP/FRT technique [19]. Recombination was induced 48–72 hours after egg laying by a 60 min heat shock at 37°C or by *eyeless* induced FLP activity. Mutant clones were marked as appropriate by the absence of GFP or β-galactosidase (β-gal) antibody staining.

The following genotypes of larvae were used for generating clones:

*hsflp/+; caps^pB1^ FRT80B/M(3)i55 ubi-GFP FRT80B*
*eyflp/+; caps^pB1^ FRT80B/arm-lacZ FRT80B*
*hsflp/+; trn^28.4^ FRT2A/ubi-GFP FRT2A*
*hsflp/+; caps^Del1^ trn^28.4^ FRT80B/M(3)i55 ubi-GFP FRT80B*
*eyflp/+; caps^Del1^ trn^28.4^ FRT80B/arm-lacZ FRT80B*

### Immunostaining

Staining of third-instar larval imaginal discs and pupal retinae was performed by standard procedures [26]. The following antibodies were used: rabbit anti-Arm (1∶100; gift from M. de la Roche); guinea pig anti-Senseless (1∶1000; gift from H. Bellen); anti-BarH1 (1∶50; gift from T. Kojima); mouse anti-Prospero (1∶50), mouse anti-Cut (1∶100) and rat anti-Elav (1∶200) (all from the Developmental Studies Hybridoma Bank at the University of Iowa); mouse anti-β-galactosidase (1∶100; Promega); rabbit anti-β-galactosidase (1∶1000; Cappel); mouse and rabbit anti-GFP (1∶200; Sigma). Guinea pig anti-Trn was used at 1∶100. Fluorescently tagged secondary antibodies came from Molecular Probes and Jackson Immunoresearch.

### Confocal Imaging and Three-Dimensional Reconstruction

For three-dimensional reconstruction, eye imaginal discs were mounted between two strips of double-sided adhesive tape, by using Fluoromount-G (Southern Biotech). Discs were analysed with a BioRad Radiance 2100 laser scanning confocal microscope. Z-series were projected for three dimensional reconstruction by using Volocity 2.5.1 software.
